# Barriers to Timely Completion of Radiation Therapy in Patients with Cervical Cancer in an Urban Tertiary Care Center

**DOI:** 10.7759/cureus.1681

**Published:** 2017-09-12

**Authors:** Justin Cohen, Amy Harper, Elizabeth M Nichols, Gautam G Rao, Pranshu Mohindra, Dana Marie Roque

**Affiliations:** 1 Department of Radiation Oncology, University of Maryland School of Medicine; 2 Deptment of Obstetrics, Gynecology, & Reproductive Sciences, University of Maryland School of Medicine; 3 Division of Gynecologic Oncology, Department of Ob/Gyn, University of Maryland School of Medicine

**Keywords:** cervical cancer, radiation therapy, treatment protraction, brachytherapy, tertiary medical center, urban health, patient navigation, socioeconomic barrier

## Abstract

Background

In 2017, there will be an estimated 12,820 women diagnosed with cervical cancer in the United States, causing an estimation of 4,210 deaths. Among U.S. women, there is a 33% greater incidence and 71% higher cervical cancer mortality in high-poverty counties when compared to low-income counties [1]. In those dispositioned to chemoradiation, treatment time of less than eight weeks is associated with compromised pelvic control. We sought to identify patient or disease characteristics and socioeconomic or psychosocial barriers that contribute to delays in treatment completion in order to formulate new policies to address these needs.

​​​​​​Methods

Cervical cancer patients treated with primary chemoradiation through the University of Maryland from 2011-2016 were identified retrospectively. Patients were placed in one of two groups: those who completed radiation treatment within 56 days, and those who failed to complete treatment within 56 days. Time to completion of radiation therapy was evaluated in relation to patient and disease variables.

​​​​Results

Forty-three patients with sufficient information for inclusion were identified. The median age was 51 years. Ten patients were stage I at diagnosis (23.3%), 16 were stage II (37.2%), 11 were stage III (25.5%) and six were stage IV (14%). Histopathology revealed squamous cell carcinoma in 37 patients (86%), adenocarcinoma in three patients (7%), mixed histology in two patients (4.7%), and neuroendocrine histology in one patient (2.3%). Twenty patients (46.5%) completed treatment within the recommended timeframe of 56 days while 23 patients (53.5%) did not. The most common reasons for a protracted treatment, or failure to complete the prescribed treatment were non-compliance/psychosocial factors (10 patients, 43.5%). Age, race, primary language, marital status, insurance, employment status, HIV status, mental health, substance abuse, tobacco use, stage at diagnosis, performance status at diagnosis, BMI (body mass index, kg/m^2^) at diagnosis, and income by zip code were not significantly associated with protracted treatment. The distance to treatment center was a significant factor (p=0.07); patients who lived closest to the treatment center were least likely to complete RT in the designated time frame. This is most likely due to the location of the treatment center, which is in the heart of an urban, low socioeconomic area.

Conclusions

More than half of all cervical cancer patients presenting to an urban tertiary care center do not complete chemoradiation therapy in the recommended timeframe. Underlying psychosocial factors are prominent. The role for patient navigation in this vulnerable population must be investigated.

## Introduction

In 2017, there will be an estimated 12,820 women diagnosed with cervical cancer in the United States, causing around 4,210 deaths [1-2]. Worldwide, it is the fourth most common cause of cancer in women, with an estimated 528,000 cases [3].

Cervical cancer disproportionally impacts individuals of low socioeconomic status (SES). A comprehensive analysis among patients with cancer in the United States in the 1990s demonstrated that there was at least a 71% higher rate of cervical cancer mortality among those with low SES, and a one-third greater incidence in high-poverty counties as compared to low-poverty counties [4]. Behavioral contributors to the development of cancer, such as smoking, diet, alcohol use, obesity, physical activity, occupational and environmental exposures, and screening often vary by SES. The analysis of socioeconomic, racial, and ethnic patterns in cancer care and treatment within medical institutions remains vital in order to identify those who may benefit from targeted interventions [5].

Multiple studies demonstrate an association with poorer outcomes if radiation treatment is prolonged [6-11]. The most recent study [10] evaluated 113 patients undergoing chemoradiation plus brachytherapy. The median time to complete treatment was 68 days. The three-year pelvic failure rate was 26% when treatment lasted more than 56 days, and 9% when treatment was completed within 56 days. The American Brachytherapy Society (ABS) Cervical Cancer Brachytherapy Task Force [12] recommends completion of radiation therapy within 56 days, and this is reflected in cooperative group trials by the Radiation Therapy Oncology Group (RTOG) and Gynecologic Oncology Group (GOG). The objective of this study was to identify patient or disease characteristics and socioeconomic or psychosocial barriers that contribute to delays in treatment completion in order to formulate new policies to address these needs.

## Materials and methods

This was an IRB-exempt investigation. Consecutive patients treated with radiation at our institution and associated satellite facilities between 2011 and 2016 were identified retrospectively. The patients were placed in one of two groups: those who completed the prescribed radiation treatment within 56 days, and those who failed to complete treatment within 56 days. If brachytherapy (BT) was not indicated, time to complete external beam radiotherapy (EBRT) was calculated. The first group included all patients who received external beam radiotherapy (EBRT) ± BT and completed treatment within 56 days. The latter group included patients who completed treatment but not in the recommended timeframe, as well as those who never completed the prescribed treatment. This group includes patients who did not receive BT when it was indicated. Descriptive statistics were applied, and these two groups were evaluated in relation to patient and disease variables, using chi-square and Mann-Whitney U tests. P-values <0.05 were considered statistically significant. Heat maps were generated using an online resource (Google Fusion Tables; https://developers.google.com/fusiontables/terms). 

## Results

Forty-three patients were identified. The median age was 51 years. Ten patients were identified as FIGO (Federation Internationale de Gynecologie et D'Obstetrique) [13] stage I at diagnosis (23.3%), 16 were stage II (37.2%), 11 were stage III (25.5%) and six were stage IV (14%) (Appendix 1). Histopathology revealed squamous cell carcinoma in 37 patients (86%), adenocarcinoma in three patients (7%), mixed histology in two patients (4.7%), and neuroendocrine histology in one patient (2.3%).

The median EBRT dose to the whole pelvis was 50.4 Gy (range: 27-50.4 Gy). Eight patients received a parametrial boost, five received a cervical boost, and nine received a nodal boost. 32 patients received BT (74.4%). 30 patients (69.8%) were treated with high-dose-rate (HDR) BT, and three (7%) received interstitial BT (one patient received both interstitial BT and HDR). The median BT dose was 28 Gy. BT was not completed due to stage IVB disease in three patients, IB1 disease status (post radical hysterectomy) in three instances, the presence of a large fibroid uterus in one patient, poor performance status due to disease burden in one patient, inability to administer appropriate anesthesia in one patient, non-compliance in one patient, and one case of patient intolerance. 

**Figure 1:**
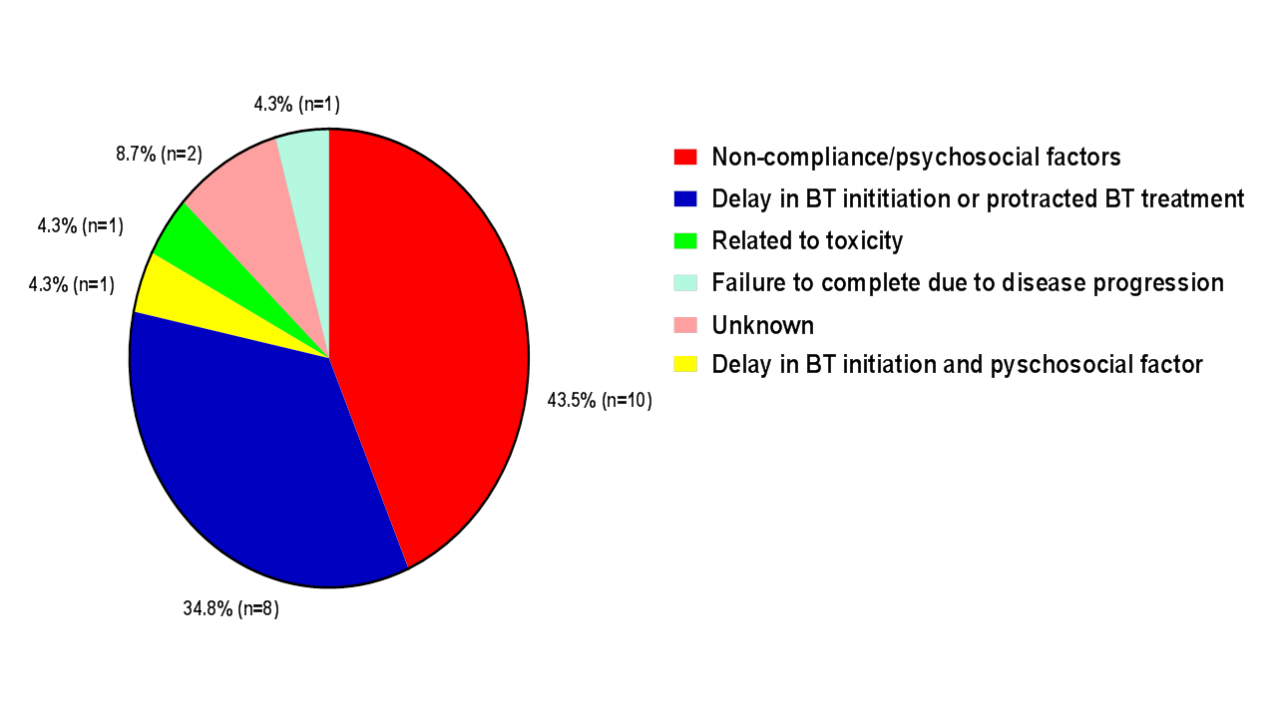
Contributors to protracted treatment.

Average time to complete RT treatment was 57 days. Twenty patients (46.5%) completed treatment within the recommended timeframe of 56 days while 23 patients (53.5%) did not. The most common reasons for a protracted treatment or failure to complete the prescribed treatment were non-compliance and/or psychosocial factors (ten patients, or 43.5% of patients who did not complete treatment within 56 days). Delays were attributed to a delay in BT initiation, or protracted BT treatment independent of non-compliance/psychosocial factors in eight patients (34.8%). At our institution, we aim for the initial phases of brachytherapy to overlap with the final external beam radiotherapy treatments, with successive brachytherapy treatment separated by at least one day. Reasons for delayed initiation sometimes stem from late referral from an outside provider, operating room scheduling conflicts that preclude timely placement of the Smit sleeve, or medical complications. Contributors to protracted BT treatment may include failure of the patient to return as scheduled for successive treatments, failure of the Smit sleeve device requiring replacement, and medical complications among others. Both a delay in BT initiation and a psychosocial factor which resulted in missed EBRT appointments occurred in one patient (4.3%). Delays were attributed to treatment-related toxicity in one patient (4.3%). One patient (4.3%) was unable to complete the prescribed treatment due to progression of disease. Retrospective chart review failed to reveal the reason for delay in two patients (8.7%) (Figure 1).

**Figure 2:**
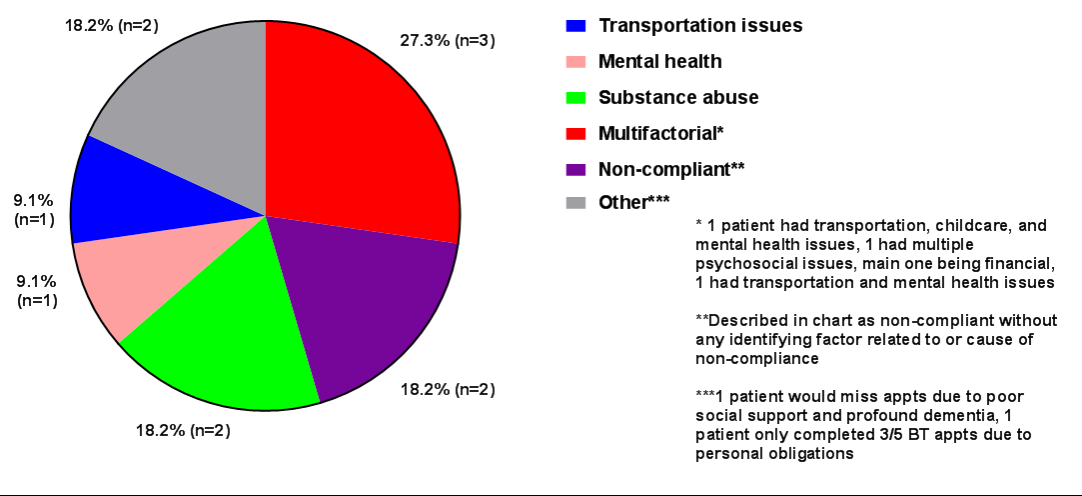
Non-compliance and psychosocial factors related to protracted treatment.

Among the psychosocial factors that contributed to delays or non-compliance were inadequate transportation in one patient (9.1% of the 11 patients with psychosocial factors), mental health in one patient (9.1%), and substance abuse in two patients (18.2%). Three patients (27.3%) had multiple factors which contributed to a delay in treatment completion. One had childcare, transportation, and mental health issues. One was described as having multiple psychosocial constraints with significant financial stressors. The third patient had financial and mental health issues. Two patients (18.2%) were labeled as being “non-compliant” without any identifiable cause documented. One patient (9.1%) missed EBRT appointments due to poor social support and profound dementia. One patient (9.1%) completed only three out of five BT appointments due to personal obligations (Figure 2).

Of the nine patients in which there was a delay in BT initiation, there were several reasons underlying the delay. The most common issue was related to tumor constraints necessitating a modification in plan from intracavitary BT to interstitial BT. Other reasons included slow or minimal response to EBRT, medical complications requiring treatment prior to BT, toxicity, or unsuccessful placement. In two patients, it was unclear why there was a delay in BT initiation.

**Figure 3:**
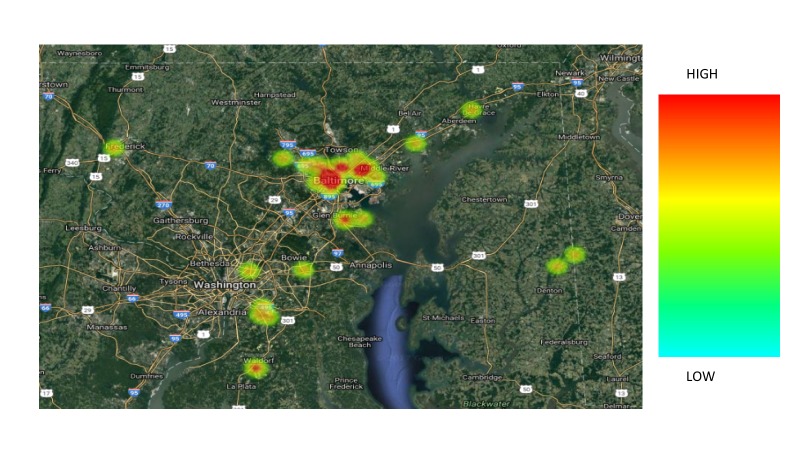
Catchment area among all patients.

**Figure 4:**
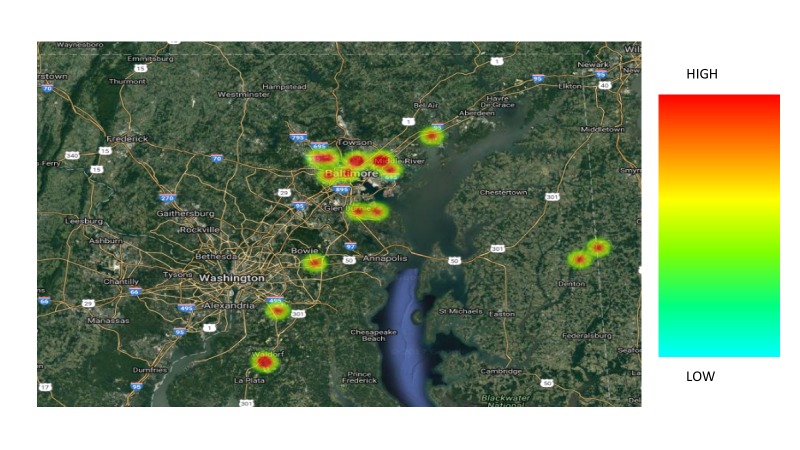
Catchment area among patients who completed treatment within 56 days.

**Figure 5:**
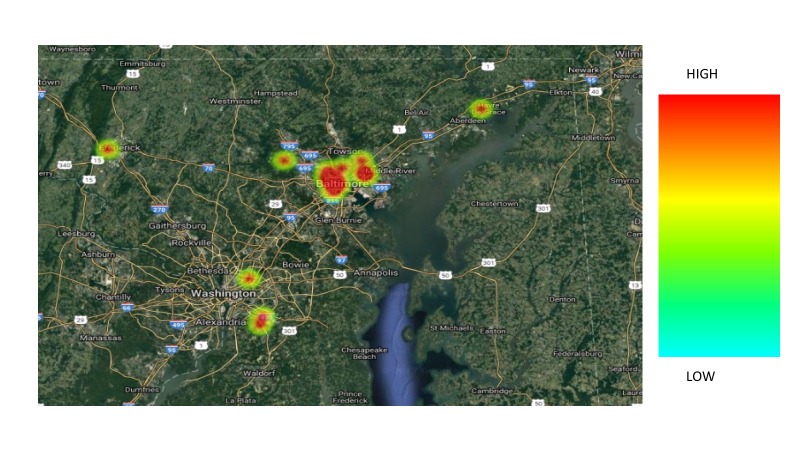
Catchment area among patients who failed to complete treatment within 56 days.

In comparing the two groups, the following variables were analyzed: age, race, primary language, marital status, insurance, employment status, human immunodeficiency virus (HIV) status, mental health, substance abuse, tobacco use, FIGO stage at diagnosis, ECOG (Eastern Cooperative Oncology Group) performance status (Appendix 2) at diagnosis, BMI (body mass index, kg/m2), diagnosis income by zip code (Table 1), and distance traveled to the hospital (Figures 3-5). None of these factors were significantly associated with a delay in treatment completion or a failure to complete treatment.

**Table 1:** Patient variables as they relate to time to complete radiation therapy. HIV - Human immunodeficiency virus Dx - Diagnosis

									≤ 56 days				> 56 days									
									*n*	*%*			*n*			*%*			*p value*			
Race																						
			White						9	45.0			5			21.7			0.18			
			Black						7	35.0			17			73.9			0.09			
			Hispanic						3	15.0			0			0.0			0.06			
			Other						1	5.0			1			4.3			0.92			
			Total						20				23									
Primary Language																						
			English						17	85.0			23			100			0.60			
			Other than English						3	15.0			0			0.0			0.06			
			Total						20				23									
Marital status																						
		Single (including divorced and widowed)							12	60.0			17			73.9			0.58			
			Married or has partner						8	40.0			6			26.1			0.43			
			Total						20				23									
Insurance																						
			No insurance						9	45.0			10			43.5			0.94			
			Medical assistance						6	30.0			9			39.1			0.61			
			Private insurance						5	25.0			4			17.4			0.59			
			Total						20				23									
Employment status																						
			Employed						11	55.0			9			39.1			0.45			
			Unemployed						8	40.0			13			56.5			0.44			
			Disability						1	5.0			1			4.3			0.92			
			Total						20				23									
HIV status																						
			Positive						1	5.0			2			8.7			0.64			
			Negative/not tested						19	95.0			21			91.3			0.85			
			Total						20				23									
Mental health																						
			Documented mental health issues						7	35.0			10			43.5			0.66			
			No documented mental health issues						13	65.0			13			56.5			0.72			
			Total						20				23									
Substance abuse																						
			Substance abuse at time of dx						3	15.0			6			26.1			0.43			
			No substance abuse at time of dx						17	85.0			17			73.9			0.68			
			Total						20				23									
Tobacco																						
			Tobacco use						11	55.0			13			56.5			0.95			
			No tobacco use						9	45.0			10			43.5			0.94			
			Total						20				23									
FIGO stage at diagnosis																						
			I						7	35.0			3			13.0			0.13			
			II						7	35.0			9			39.1			0.81			
			III						3	15.0			8			34.8			0.19			
			IV						3	15.0			3			13.0			0.86			
			Total						20				23									
ECOG score at diagnosis																						
			0						13	65.0			9			39.1			0.24			
			1						5	25.0			12			52.2			0.16			
			2						1	5.0			2			8.7			0.64			
			3						0	0			0			0			N/A			
			4						1	5.0			0			0			0.28			
			Total						20				23									
	≤56 days							>56 days														
	*mean {range}*					*SD*	*SEM*	*mean {range}*				*SD*			*SEM*			*p value*				
Age (years)	51.28 {36-65}					7.8	1.8	49.75 {26-92}			17.2			3.6			0.86					
BMI (kg/m^2^)	29.75 {19.0-47.7}					8.5	1.9	27.81{18.5-62.9}			11.7			2.4			0.18					
Distance to hospital (miles)	23.48 {0.3-72.1}					25.6	5.9	14.15 {0.3-50.8}			16.5			3.4			0.07					
Income by zip code ($)	63,367 {30,305-117,355}					22,592	5,183	55,727 {30,121-78164}			15,129			3,154			0.25					

 

## Discussion

Individuals of low SES may have some unique barriers that prevent the timely completion of cancer therapy [14-15]. Many patients require assistance with coordination of care and navigation of a complex healthcare system. We sought to identify barriers as a target for intervention to improve compliance with therapy, and decrease the amount of time to complete RT for patients with cervical cancer receiving care at an urban tertiary care center.

We investigated age, race, primary language, marital status, insurance coverage, employment status, HIV status, mental health diagnoses, substance abuse, tobacco use, FIGO stage at diagnosis, ECOG performance status at diagnosis, BMI at diagnosis, distance to treatment center, and income by zip code. Distance to treatment center approached significance and deserves further investigation as a characteristic of those who were unable to complete therapy within 56 days, with a p-value of 0.07. Interestingly, patients who lived the closest to the treatment center were least likely to complete RT in the designated timeframe. This is most likely due to the location of the treatment center, which is in the heart of an urban, low SES area. None of these factors, however, proved to significantly impact the timely completion of therapy.

Of the study participants who did not complete RT treatment within the recommended 56 days, approximately 48% had psychosocial factors contributing to delay in therapy or non-compliance. Investigation of the chart documentation revealed that the major barriers to care in this group were transportation, child care, mental health issues, and substance abuse. This suggests that the approach to treatment for these patients requires a multi-disciplinary team that can provide the individualized support necessary to complete demanding treatment protocols. A patient navigator to evaluate patients beginning to fall behind in their treatment schedule would be a beneficial resource to identify specific barriers and psychosocial factors contributing to delay in treatment, and a means of support for these patients.

Patient navigation can play an important role in survivorship among cancer patients. It has been proven to be an effective method of addressing barriers to care for patients of low SES. Several studies have suggested that patient navigation improves timeliness of treatment and compliance with follow-up care, specifically in low SES groups. Patient adherence to follow-up care has been estimated to increase by approximately 29% with the addition of a patient navigator to the care team [16]. Patient navigators not only aid with transportation, care coordination, and obtaining health insurance, but also serve as a social support and can bolster rapport between the treatment team and the patient [17]. Gorin, et al. published data of a systematic review and meta-analysis of cancer care coordination from 1980-2015 in various cancer sites. The most common form of cancer care coordination was patient navigation. The authors reported an 81% improvement of outcomes with patient navigation, including screening, measures of patient experience with care, and quality of end-of-life care [18]. In cervical cancer, patient navigation has mostly been employed in screening settings [19-21], and only rarely to treatment settings [22].

A common factor among those with protracted RT courses was psychosocial stressors that are common to low SES populations. Transportation, childcare, mental health issues, and substance abuse were all specific issues identified in contributing to delays in completion of RT therapy. The incorporation of a patient navigator could aid in identifying and overcoming such barriers to care to improve compliance with RT treatment schedules and follow-up.

Small sample size was the largest limitation of the present study. We hope to continue to engage participants and prospectively study the role of patient navigation in affecting timely completion of RT. Even within the small study population, the impact of psychosocial factors among those who failed to complete RT within 56 days was evident.

## Conclusions

More than half of all cervical cancer patients presenting to an urban tertiary care center do not complete chemoradiation therapy in the recommended timeframe. Underlying psychosocial factors are prominent. The role for patient navigation in this population must be further investigated, and may play an important role in the cases of vulnerable cervical cancer patients.

## Appendices

APPENDIX 1: FIGO (Federation International de Gynecologie et D'Obstetrique) Cervical Cancer Staging

IA1: Invasion of stroma no greater than 3 mm in depth and no wider than 7 mm

IA2: Invasion of stroma greater than 3 mm, no greater than 5 mm in depth and no wider than 7 mm

IB1: Clinically apparent lesion confined to the cervix greater than or equal to 4 cm

IB2: Clinically apparent lesion confined to the cervix less than 4 cm

IIA: Disease extends beyond the cervix but has not extended to pelvic sidewall or lower third of vagina, no parametrial invasion

IIB: Disease extends beyond the cervix but has not extended to pelvic sidewall or lower third of vagina, with parametrial invasion

IIIA: Disease extends beyond the cervix but has not extended to pelvic sidewall, involves lower third of vagina

IIIB: Disease extends to pelvic sidewall and/or hydronephrosis, or non-functioning kidney

IVA: Bladder or bowel involvement

IVB: Distant metastases

APPENDIX 2: Eastern Cooperative Oncology Group Performance Status

0: Fully active, able to carry on all pre-disease performance without restriction

1: Restricted in physically strenuous activity but ambulatory, and able to carry out work of a light or sedentery nature 

2: Ambulatory and capable of all selfcare, but unable to carry out any work activities

3: Capable of only limited self-care, confined to bed or chair for more than 50% of waking hours

4: Completely disabled, unable to perform any self-care, totally confined to bed or chair

5: Dead
